# Dysregulation of Rho‐Associated Coiled‐Coil Protein Kinase1 Depletes Neural Stem Cell Pool and Impairs Hippocampal Neurogenesis After Traumatic Brain Injury

**DOI:** 10.1111/cpr.70093

**Published:** 2025-08-01

**Authors:** Chaoqun Yao, Long Jin, Jun Zhong, Qianying Huang, Zhongwei Bao, Shaolong Zhou, Chaohua Wang, Huanhuan Li, Xiaowei Yuan, Zhen Wang, Ning Du, Jingxuan Yu, Huanran Chen, Xuyang Zhang, Hongfei Ge, Jianheng Wu

**Affiliations:** ^1^ Department of Neurosurgery and Key Laboratory of Neurotrauma, Southwest Hospital Third Military Medical University (Army Medical University) Chongqing China; ^2^ Department of Neurosurgery The Fifth Affiliated Hospital of Zhengzhou University Zhengzhou China; ^3^ Clinical Medical Research Center, Southwest Hospital Third Military Medical University (Army Medical University) Chongqing China

**Keywords:** AKT, cognitive impairment, neurogenesis, ROCK1, traumatic brain injury

## Abstract

Traumatic brain injury (TBI) represents a global health burden, often resulting in persistent neurological deficits due to impaired hippocampal neurogenesis. Nevertheless, the temporal progression of post‐TBI neurogenesis and its molecular mechanisms remain elusive. To investigate the mechanism of impaired hippocampal neurogenesis and neurological deficits following TBI. Single‐cell RNA sequencing (scRNA‐seq) was employed to explore the mechanism of abnormal hippocampal neurogenesis after TBI in mice. Antagonists and conditional gene knockout (CKO) strategies were applied to dissect the molecular function of target genes. Here, we found that neural stem cells (NSCs) were hyperactivated as observed in Nestin‐GFP reporter mice in hippocampus during the early phases of TBI, followed by progressive depletion of the NSC pool, impaired neurogenesis, and the onset of progressive cognitive dysfunction. ScRNA‐seq transcriptomic analysis revealed sustained upregulation of Rho‐associated coiled‐coil protein kinase 1 (ROCK1) in hippocampal NSCs post‐TBI. Pharmacological inhibition of ROCK1 or ROCK1 CKO rescued chronic neurogenic deficits and improved cognitive functions in TBI mice. Mechanistically, ROCK1 dysregulation impaired neurogenesis via aberrant AKT hyperphosphorylation, establishing a unidirectional ROCK1‐AKT signalling axis in adult hippocampal neurogenesis. Our findings position ROCK1 as a pivotal regulator of the post‐TBI NSC pool hyperactivation and aberrant neurogenesis and propose targeted kinase inhibition strategies as a potential therapy to mitigate abnormal neurogenesis in TBI patients.

## Graphical abstract

Following TBI, hippocampal neurogenesis in mice is impaired and affects cognitive function. This impairment is associated with the upregulation of p‐AKT/AKT mediated by ROCK1.

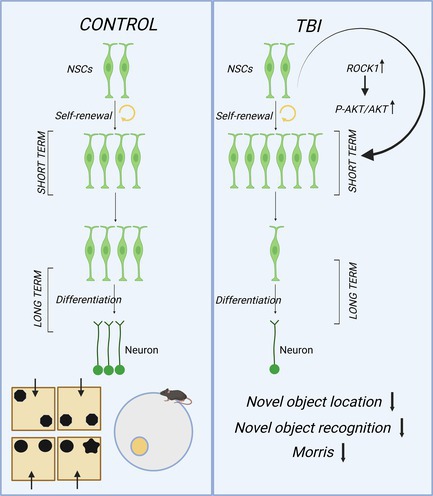

## 1 Introduction

Traumatic brain injury (TBI) affects approximately 69 million individuals globally each year [1], and represents a pervasive public health challenge. Diverse etiological factors, including vehicle collisions, sports‐related impacts, and occupational hazards [2, 3], can cause TBI pathology. TBI pathophysiology involves both primary mechanical shock‐induced damages as well as secondary physical injuries encompassing neuronal and vascular injuries, demyelination and reactive gliosis [4, 5].

Adult neurogenesis in the hippocampus mainly occurs in the dentate gyrus (DG), where neural stem cells (NSCs) undergo self‐renewal, differentiation, and maturation processes to maintain cognitive functions [6, 7, 8]. Under homeostatic conditions, quiescent NSCs intermittently differentiate into specific neuronal and astrocytic subtypes that integrate into the respective hippocampal circuits, supporting learning and memory processes [9, 10, 11]. Previously, we have shown that NSCs are overactivated in the hippocampal region following an acute intracerebral haemorrhagic (ICH) stroke, leading to the depletion of brain NSC pools. Notably, inhibition of the NSC pool activation facilitates neurogenesis in the hippocampal DG during chronic injury, thereby rescuing neurological and cognitive deficits following ICH [12]. Studies demonstrate that TBI enhances hippocampal neurogenesis while decreasing astrogliosis on day 14 post‐TBI, suggesting an essential contribution of hippocampal neurogenesis homeostasis to the pathogenesis of central nervous system (CNS) disorders [2]. However, the nature of dynamic changes in the NSC pool and post‐TBI neurogenesis patterns, as well as their implications in stroke pathophysiology, remain inconclusive.

Here, we exploited a mouse model of TBI to uncover the dynamic changes in the hippocampal NSC pool and the activation of neurogenesis in response to brain injuries. Through single‐cell RNA sequencing (scRNA‐seq) analysis and mechanistic validations, we found that Rho‐associated coiled‐coil protein kinase 1 (ROCK1)‐mediated dysregulated activation of Protein Kinase B (AKT) following TBI could drive excessive proliferation of NSCs in the early phase and impair neurogenesis in the late phase, ultimately contributing to heightened cognitive dysfunctions. These results further support our hypothesis that TBI may attenuate long‐term neurogenesis in the hippocampal DG, where ROCK1 plays a crucial regulatory role. Moreover, this study deepens our basic understanding that TBI might impact long‐term neurogenesis in DG, providing a feasible intervention time point and target for treating TBI, as well as related CNS injuries.

## 2 Materials and Methods

### 2.1 Mouse Strains

The mouse strains used in this paper included as follows: C57BL/6J, Nestin‐GFP, Nestin‐CreER^T2^, Rosa26R‐CAG::tdTomato, and ROCK1^flox/flox^. All animals were male; therefore, sex differences were not a factor in the experimental results. To label endogenous neural stem cells (NSCs), a crossbreeding strategy was employed between Nestin‐CreER^T2^ and Rosa26R‐CAG::tdTomato mice. For tracing NSCs in ROCK1 conditional knockout models, the initial step involved mating Rosa26R‐CAG::tdTomato reporter mice with ROCK1^flox/flox^ mice. Subsequently, the resulting offspring were crossed with Nestin‐CreERT2 mice to produce triple‐transgenic animals (Rosa26R‐CAG::tdTomato; ROCK1^flox/flox^; Nestin::CreERT2). All experimental mice were genetically stabilised through at least six generations of backcrossing onto the C57BL/6J genetic background. For genetic recombination, mice received tamoxifen injections prior to 8 weeks of age. Following tamoxifen induction, a controlled cortical impact (CCI) model of TBI was generated. Post‐injury assessments were conducted at 7 and 60 days to evaluate neurogenic and behavioural outcomes. The animals were maintained in controlled environmental conditions featuring a 12‐h photoperiod, standardised temperature, regulated humidity, and semi‐sterile housing. All procedures strictly adhered to China's animal research welfare regulations and received formal approval from Army Medical University's institutional animal care committee (approval NO. AMUWEC2020777).

### 2.2 Animal Model

For traumatic brain injury, a controlled cortical impact model was used [13, 14, 15]. Animals were randomly assigned to experimental groups using computer‐generated randomisation tables (block size = 6), with group allocation concealed until intervention completion. Mice were anaesthetised with 5% isoflurane, then horizontally fixed on a stereotactic frame and maintained under 2% isoflurane. The hair and the skin were incised to expose the skull. A craniotomy of approximately 4 mm in diameter was performed to expose the cerebral cortex. The controlled cortical impact (CCI) procedure was performed using a stereotactic impactor (Leica Biosystems, Buffalo Grove, IL, USA) attached to the stereotactic frame. The impact parameters were: speed, 4 m/s; residence time, 200 ms; and depth, 1.5 mm. After the impact, the skin was closed with monofilament sterile sutures, and the animals were administered 0.9% sodium chloride solution (IP). The animals were then transferred to a recovery cage until full recovery of movement.

### 2.3 Western Blot

WB samples were microdissected from hippocampal DG. For tissue lysis, hippocampus tissue was homogenised with RIPA lysate. Proteins were separated by SDS‐PAGE on a 10% polyacrylamide gel under reducing conditions and electroblotted onto polyvinylidene difluoride (PVDF) membranes. Membranes were blocked with 5% non‐fat milk in TBST for 2 h at room temperature, washed three times with TBST (10 min per wash), and incubated overnight at 4°C with gentle shaking with the following primary antibodies: anti‐ROCK1 (1:1000, 21,850‐1‐AP, Proteintech), anti‐phospho‐Akt (1:2000, 66,444‐1‐Ig, Proteintech), anti‐Akt (1:1000, 60,203‐2‐Ig, Proteintech), anti‐GAPDH (1:10,000, sc‐32,233, Santa Cruz Biotechnology), and anti‐β‐actin (1:10000, Boster). Membranes were washed with TBST, followed by incubation with HRP‐conjugated anti‐rabbit and anti‐mouse IgG secondary antibodies (1:5000, CST) for 2 h at room temperature. Protein bands were detected using enhanced chemiluminescence (ECL) substrate (P0018, BeyoECL Plus, Beyotime) and imaged with a ChemiDoc XRS+ system (Bio‐Rad). Band intensities were quantified using Image Lab software (version 2.0.1, Bio‐Rad).

### 2.4 Immunofluorescence

For immunofluorescence staining, 30‐μm‐thick brain cryosections were permeabilised with 0.5% Triton X‐100 in PBS for 30 min, followed by blocking in 5% bovine serum albumin (BSA)/PBS diluted in PBS containing 0.1% Tween‐20 (PBST) for 1 h at room temperature. The sections were then incubated overnight at 4°C with primary antibodies diluted in blocking buffer. After washing three times with PBS, sections were incubated with species‐matched Alexa Fluor‐conjugated secondary antibodies for 2 h at room temperature in the dark. Nuclei were counterstained with 4′,6‐diamidino‐2‐phenylindole (DAPI; 1 μg/mL) for 10 min. Finally, coverslips were mounted using antifade mounting medium, and images were acquired using a Zeiss LSM 880 confocal microscope (Carl Zeiss, Jena, Germany) equipped with ZEN imaging software (version 3.0; Carl Zeiss). Primary antibodies used in this study were as follows: rabbit anti‐Sox2 (1:100, abcam, 97,959), mouse anti‐GFAP (1:200, CST, 3670), rabbit anti‐GFAP (1:400, CST, 12389), GFP (1:1000, Aves labs,1020), GFP (1:500, Invitrogen, 10,262), rabbit anti‐Ki67 (1:400, CST, 9129), rabbit anti‐DCX (1:500, CST, 4604), rabbit anti‐NeuN (1:500, CST, 24309). Secondary antibodies were applied at 1:500 dilution(goat anti‐chicken Alexa Fluor 488, ab150173, donkey anti‐rabbit Alexa Fluor 488, ab150173, goat anti‐rabbit Alexa Fluor 555, ab150078, goat anti‐mouse Alexa Fluor 555, ab150118, goat anti‐rabbit Alexa Fluor 647, ab150079, goat anti‐mouse Alexa Fluor 647, ab150115).

### 2.5 Behavioural Tests

Novel Object Location Test (NOL), Novel Object Recognition Test (NOR) and Morris water maze (MWM) were used in the experiments to evaluate cognitive function of mice [16, 17].

NOL and NOR: On day 1, mice were allowed to freely explore the arena for 10 min. After 24 h, two identical objects were put about 5 cm away from the wall on the side of the arena. Mice were allowed to move freely for 15 min. After all the mice were familiar with the objects, one of the objects was transferred to the opposite side or one of the original objects was replaced with a new object. The distance from the object to the wall was the same. Mice were again placed in the arena and were allowed to explore for 10 min. The exploratory preference was calculated as the percentage of time spent investigating the opposite side object or new object in total time spent exploring objects.

Morris water maze: The experiments were conducted in a circular water maze. The escape platform remained fixed at a constant spatial position within the pool. During the training phase, the platform was submerged 1 cm below the water surface. Mice were sequentially introduced into the maze from four distinct starting points (north, south, east, west) in a randomised order. Each animal underwent four consecutive trials daily over a six‐day training period. Twenty‐four hours after the final training session, the platform was removed, and swimming trajectories of all mice were recorded for spatial memory analysis.

### 2.6 Stereotactic Injection of Viruses

The adeno‐associated virus (AAV, viral titre 8.3108/mL, injection volume 300 nL) were injected into the dentate gyrus of Nestin‐Cre mice to achieve a conditional loss of AKT in hippocampal neural stem cells. To ensure viral infection in the dentate gyrus of the hippocampus, multi‐site injections were applied. The first coordinate point represents the displacement of 2.0 mm posterior, 1.4 mm lateral, and 1.64 mm in depth. The second coordinates are the reference points of 2.0 mm at the front, 1.9 mm on the side, and a depth displacement of 2.0 mm. The mice were prepared for the analysis after 21 days of the infusion.

### 2.7 EdU Administration

To label proliferative cells in vivo, EdU(Beyotime, ST067)was dissolved in PBS and injected intraperitoneally 24 h before experimental analysis at a dose of (50 mg/Kg). For labeling differentiated cells in vivo, EdU was dissolved in PBS and injected intraperitoneally 30 days before experimental analysis at a dose of (200 mg/Kg).

### 2.8 Tamoxifen Administration

To induce recombination, 7‐week‐old mice were intraperitoneally injected with Tamoxifen (Sigma‐Aldrich, T5648) once a day for 5 consecutive days. The dosage was 100 mg per kilogram of body weight. The solvent of Tamoxifen was a mixture of 10% ethanol and sesame oil.

### 2.9 ARQ092 and Y27632 Administration

To inhibit the phosphorylation of AKT in vivo, ARQ092 (HY‐19719, MedChemExpress, USA) dissolved in DMSO and normal saline was administered to mice at a dose of 200 mg/Kg. To inhibit Rock1 in vivo, Y27632 (SC0326–5 mg, Beyotime, China) dissolved in DMSO and normal saline was intraperitoneally injected into mice at a dose of 10 mg/Kg. The doses were referred to the instructions.

### 2.10 Fluorescence‐Activated Cell Sorting

GFP+ NSCs were isolated from the dentate gyrus of Nestin‐GFP mice using a BD FACSAria III cell sorter (BD Biosciences) equipped with a 100‐μm nozzle. Single‐cell suspensions were prepared by enzymatic dissociation with papain (20 U/mL; Worthington) and filtered through 40‐μm cell strainers (Falcon). Propidium iodide (PI; 1 μg/mL; Sigma) was used for live/dead discrimination, detected in the 670/14 nm channel (APC‐Cy7 filter set). The hierarchical gating strategy included: (1) Debris exclusion: Threshold set on forward scatter (FSC‐A > 5 × 10^2^) and side scatter (SSC‐A < 2 × 10^3^). (2) Doublet discrimination: FSC‐H vs. FSC‐W analysis excluding events beyond the linear correlation. (3) Viable cell selection: PI‐negative population (FL4 < 10^3^). (4) GFP+ sorting: FITC channel detection (530/30 nm) with compensation for spectral overlap using single‐stained controls. Sorted GFP^+^ cells (purity > 95% verified by post‐sort reanalysis) were collected in RNAlater solution (Thermo Fisher) for subsequent RT‐qPCR analysis. All procedures complied with MIFlowCyt reporting standards.

### 2.11 RT‐qPCR

We extracted total RNA from animal cells using Trizol (Invitrogen) according to the manufacturer's instructions. The cDNA was reverse transcribed by the ezDNase enzyme kit (Promega). RT‐qPCR was performed using the QuantStudio Real‐time PCR system (Applied Biosystems). Primers used for qPCR are as follows:

Rock1:

Forward primer::5′‐GCTCATCTCTGTGTGACTCT‐3′.

Reverse primer:5′‐TACGGAAAGCAAGTCAGACC‐3′.

PROX1:

Forward primer::5′‐AAAGTCAAATGTACTCCGCAAGC‐3′.

Reverse primer:5′‐CTGGGAAATTATGGTTGCTCCT‐3′.

MAP1b:

Forward primer:: 5′‐CCTCAGATGTGGGTGGCTATTA‐3′.

Reverse primer:5′‐TGTCTTGGGGTGCTTGTATTCA‐3′.

PTBP1:

Forward primer::5′‐GTGTGCCATGGACGGCATT‐3′.

Reverse primer:5′‐TTTGCTGCAGAAGCCGAGT‐3′.

SOX11:

Forward primer:5′‐CGAGCCTGTACGACGAAGTG‐3′.

Reverse primer:5′‐AAGCTCAGGTCGAACATGAGG‐3′.

GSK3B:

Forward primer:5′‐GGTGGCTCTTTGTTTGCCTG‐3′.

Reverse primer:5′‐TCAGCCTTTGACCTCAGCAG‐3′.

RTN4:

Forward primer:5′‐CTCCTCTGGTCTCGTCCTC‐3′.

Reverse primer:5′‐GTCCTCGTCCTCCTCTTCC‐3′.

AKT:

Forward primer:5′‐AGGAGGAAGAGATGATGGAT‐3′.

Reverse primer:5′‐GAATGGATGCCGTGAGTT‐3′.

GAPDH:

Forward primer:5′‐AACGGGAAGCCCATACC‐3′.

Reverse primer: 5′‐CATACTCAGCACCGGCCTCA‐3′.

### 2.12 Analysis of the Single‐Cell Sequencing Data

Data analysis was performed using Seurat (version 5.0.1). Cells underwent t‐SNE dimension reduction using the RunTSNE function, and the dimension reduction results were visualised. Cell types were annotated based on known marker genes. Gene expression of NSC cells was normalised using the SCTransform function of Seurat. The normalised NSC cells were t‐SNE dimension reduced using the RunTSNE function, and the cells were clustered using the FindClusters function (resolution 0.2). The clustering results were annotated with cell types based on known marker genes, and the proportion of cell numbers in different experimental groups was analysed. The differential expression of genes in each NSC subtype between the TBI group and the Sham group was calculated using the FindMarkers function. Significantly differentially expressed genes (with avg_log2FC > 1 and *p*_val < 0.05) were identified and subjected to GO Biological Process pathway enrichment analysis using cluster Profiler (version 4.8.3). Enrichment results were visualised using ggplot2 (version 3.4.3). All analyses were performed within the R environment (version 4.3.1).

### 2.13 Statistical Analysis

All data were analysed using SPSS 23.0 and presented as mean ± SEM. All behavioural assessments, histological analyses, and data quantification were performed by investigators blinded to group allocation. Treatment codes were maintained by an independent statistician until final analysis. The distribution of data was verified with the Shapiro–Wilk test. Independent‐sample t‐tests were used for numerical comparisons between the two groups, while one‐way ANOVA was used for comparisons between multiple groups, and tested with Turkey's post hoc test. *p* < 0.05 was considered statistically significant.

## 3 Results

### 3.1 TBI Over‐Activates Hippocampal NSC Pool in the Acute Phase and Decreases Neurogenesis

**FIGURE 1:**
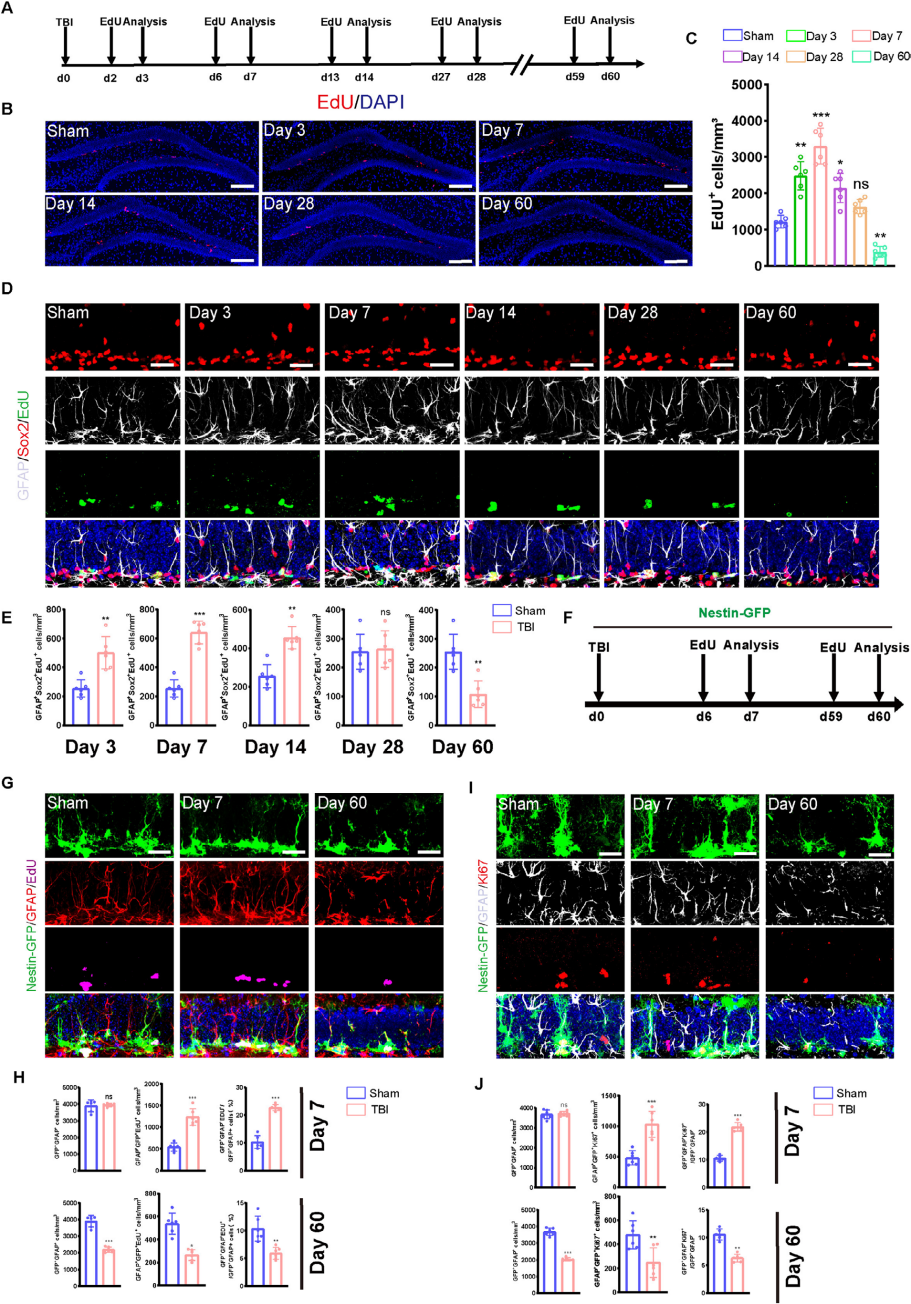
Abnormal proliferation of hippocampal neural stem cells after traumatic brain injury. (A) Schematic of the experimental timeline: EdU injection and tissue collection for proliferation analysis in WT mice. (B) Representative micrographs of EdU‐labelled proliferating cells in the DG of TBI mice harvested at 3, 7, 14, 28, and 60 days post‐injury. Scale bar = 100 μm. (C) Quantification of numbers of proliferating cells (EdU^+^) from B. (*n* = 6). *p < 0.05；**p < 0.01, ns, not significant. One‐way ANOVA followed by Tukey's post hoc test. (D) Representative images of brain sections immunostained for Sox2 and GFAP, and labelled with EdU, showing the DG in TBI mice at 3, 7, 14, 28, and 60 days post‐injury. Scale bar = 20 μm. (E) Quantification of numbers of proliferating neural cells (GFAP^+^Sox2^+^EdU^+^) from B. (*n* = 6). (F) The experimental timeline for EdU administration and cell proliferation analysis in Nestin‐GFP mouse. (G) Representative images of brain sections immunostained for GFAP, labelled with EdU, and showing genetically expressed GFP in the DG of TBI mice at 7 and 60 days post‐injury. Scale bar = 20 μm. (H) Quantification of proliferating rNSCs (GFP^+^GFAP^+^EdU^+^) total NSCs (GFP^+^GFAP^+^) and the proportion of newly proliferated NSCs among total NSCs in group G (*n* = 6). (I) Representative images of the DG in TBI mice at 7 and 60 days post‐injury, showing proliferating neural stem cells (Ki67^+^GFAP^+^GFP^+^ cells). Scale bar = 20 μm. (J) Quantification of numbers of rNSCs which enter cell cycle (GFP^+^GFAP^+^Ki67^+^) cells from I. (*n* = 6). *p < 0.05, **p < 0.01, ***p < 0.001; ns, not significant, Student's *t* test.

NSCs have the potential to self‐renew and differentiate into neurons and glial cells through neurogenesis [7, 11, 18, 19]. Previous studies have only reported TBI‐induced altered hippocampal NSC pool dynamics up to Day 14. To address this limitation, we established a mouse model of TBI to examine hippocampal tissues on days 3, 7, 14, 28, and 60 post‐TBI. Mouse brain proliferating NSCs were labelled by intraperitoneal (i.p.) injection of EdU 24 h before euthanasia (Figure 1A), revealing a significant increase in the populations of EdU^+^ hippocampal NSCs on days 3, 7, and 14. Interestingly, the number of EdU^+^ cells was the same as that in the sham group on day 28; however, it decreased significantly on day 60 compared to the sham group (Figure 1B,C). Furthermore, we found that populations of GFAP^+^/Sox2^+^/EdU^+^ radial NSCs (rNSCs) were significantly increased in the hippocampus on days 3, 7, and 14 days post‐TBI. On day 28, the population of proliferating rNSCs in the TBI group showed no significant difference from the sham group; however, on day 60, the population of this NSC subtype decreased significantly compared to the sham group (Figure 1D,E). Next, we exploited a Nestin‐GFP transgenic reporter mouse line to dynamically monitor the proliferation of NSCs following TBI (Figure 1F). We found that TBI induced a significant increase in the population of hippocampal proliferating rNSCs (GFAP^+^/GFP^+^/EdU^+^) on day 7 but decreased on day 60 compared to the sham‐operated group. Additionally, the neural stem cell pool (GFAP^+^/GFP^+^) showed no significant change on day 7 post‐TBI but became depleted by day 60. Further analysis revealed that the proportion of newly proliferated neural stem cells (GFAP^+^/GFP^+^/EdU^+^) among total NSCs increased on day 7 post‐TBI but decreased on day 60 (Figure 1G,H). We also noticed a similar trend in the population of GFAP^+^/GFP^+^/Ki67^+^ cells in the hippocampus on day 7 versus day 60 post‐TBI, suggesting that acute phase TBI promotes cell cycle entry of quiescent NSCs (Figure 1I,J). Taken together, these results indicate that NSCs could be overactivated in the early stages of TBI, leading to a putative premature exhaustion of the hippocampal NSC pools.

### 3.2 TBI Impairs Hippocampal Neurogenesis and Induces Cognitive Dysfunction

**FIGURE 2:**
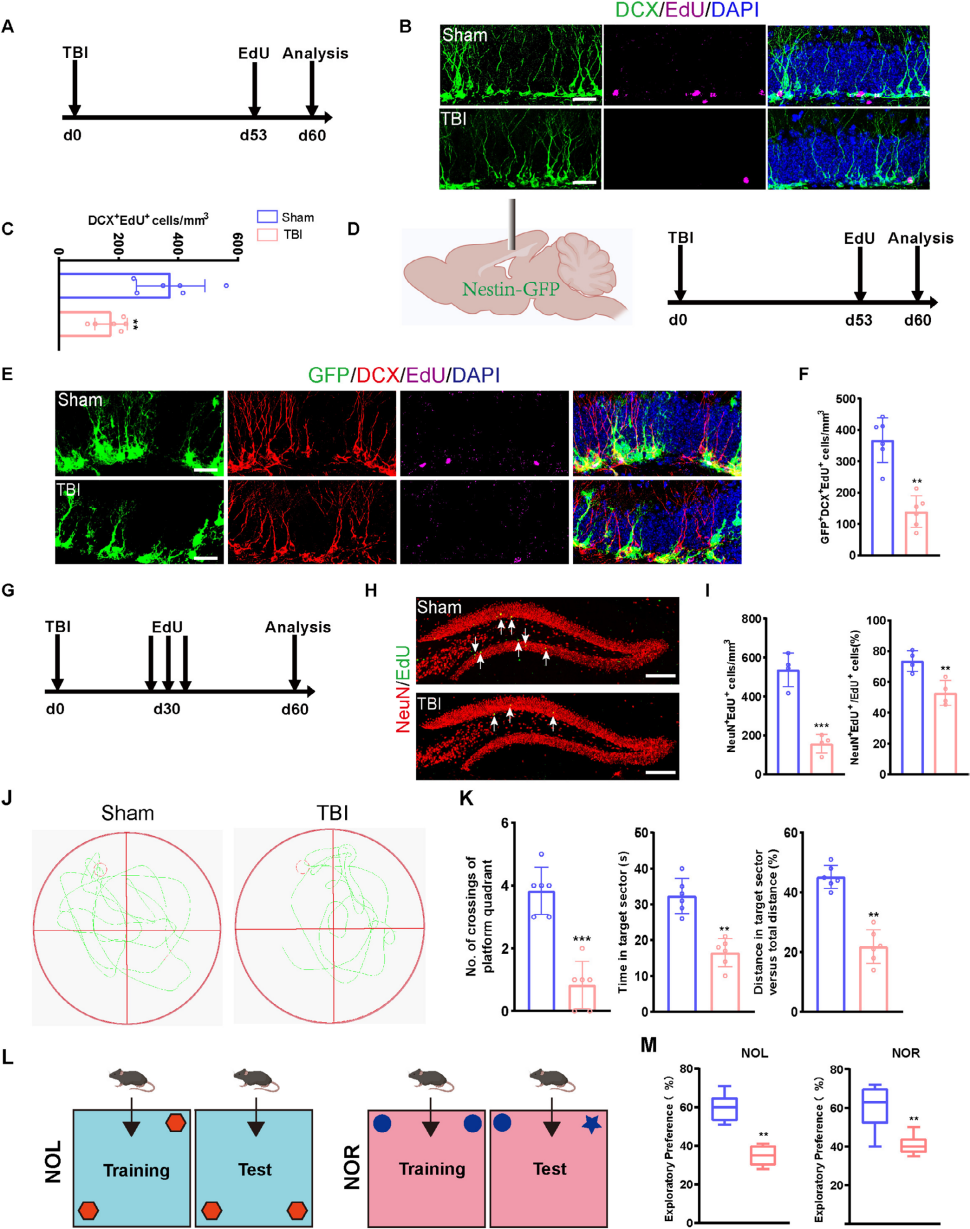
Long‐term neurogenesis is impaired and cognitive decline occurs after traumatic brain injury. (A) The timeline for EdU administration and cell differentiation analysis in WT mice. (B) Representative images of DCX^+^ immature neurons and EdU‐labelled proliferating cells in the DG of TBI mice at 60 days post‐injury. Scale bar = 20 μm. (C) Quantification of numbers of new‐born immature neural cells (DCX^+^EdU^+^) from B (*n* = 6). (D) The experimental timeline for EdU administration and cell differentiation analysis in Nestin‐GFP mouse. (E) Representative images of brain sections immunostained for DCX, labelled with EdU, and showing genetically expressed GFP in the DG of TBI mice at 60 days post‐injury. Scale bar = 20 μm. (F) Quantification of newborn immature neurons (GFP^+^DCX^+^EdU^+^) derived from GFP^+^ progenitor cells in group E (*n* = 6). (G) The experimental timeline for EdU administration and cell differentiation analysis in WT mouse. (H) Representative images of brain section stained with NeuN, labelled with EdU in the DG of TBI mice at 60 days post‐injury. Scale bar = 100 μm. (I) Quantification of new‐born mature neurons (NeuN^+^EdU^+^) in G (*n* = 4). (J) Representative swim paths of Sham‐operated and TBI mice during the probe trial in the Morris water maze at 60 days post‐injury. (K) Behavioural performance of Sham‐operated and TBI mice in the Morris water maze: Escape latency during acquisition training; Time spent in the target quadrant during the probe trial; Number of crossings over the former platform location (*n* = 6). (L) Schematic diagram of novel object location and novel object recognition test. (M) TBI mice exhibited reduced exploratory preferences in the NOL test (N) and NOR test. (*n* = 6). *p < 0.05, **p < 0.01, ***p < 0.001; ns, not significant, Student's *t* test.

Building on these findings, we employed EdU pulse‐chase labeling to quantitatively evaluate the spatiotemporal dynamics of hippocampal neurogenesis following TBI. EdU was administered on day 7 to label newly differentiated pre‐mature neurons (Figure 2A,D), which showed that the population of DCX^+^/EdU^+^ NSCs was significantly reduced in the TBI group compared to the sham‐operated control group (Figure 2B,C). Additionally, we also noticed that the population of GFP^+^/DCX^+^/EdU^+^ NSCs was significantly decreased in the Nestin‐GFP group compared to the sham group (Figure 2E,F). Furthermore, to assess whether TBI‐induced NSC pool overactivation impacts neurogenesis in the long term, we administered EdU daily for 3 consecutive days to track the status of proliferating NSCs through day 60 after TBI (Figure 2G). Our results also indicate that the population of hippocampal NeuN^+^/EdU^+^ NSCs was significantly lower in the TBI than in the sham‐operated group on day 60. Besides, we found that the number of newborn mature neurons was significantly lower in the TBI group than in the sham group (Figure 2H,I), suggesting impaired hippocampal neurogenesis in TBI mice.

Cognitive impairment is a common complication of TBI [20, 21]. Cognitive and memory functions heavily rely on the dynamics of synaptic communications through neurogenesis [22, 23, 24, 25]. To evaluate cognitive deficits in TBI mice, we performed MWM, NOL, and NOR tests on day 60 post‐TBI. In the MWM test, TBI mice spent significantly less time in the target quadrant, crossed the platform locations fewer times, and travelled shorter distances compared to sham mice (Figure 2J,K), indicating impaired spatial memory. Similarly, NOR and NOL tests revealed significantly reduced preferences for the novel object in the TBI group compared to the sham group (Figure 2L–N), suggesting deficits in recognition memory. These behavioural impairments might have resulted from disrupted hippocampal neurogenesis, as evidenced by the decreased number of newborn mature neurons in TBI mice.

### 3.3 ScRNA‐Seq Analysis Identifies ROCK1 as a Critical Player in the Post‐TBI Impairment of Neurogenesis

**FIGURE 3:**
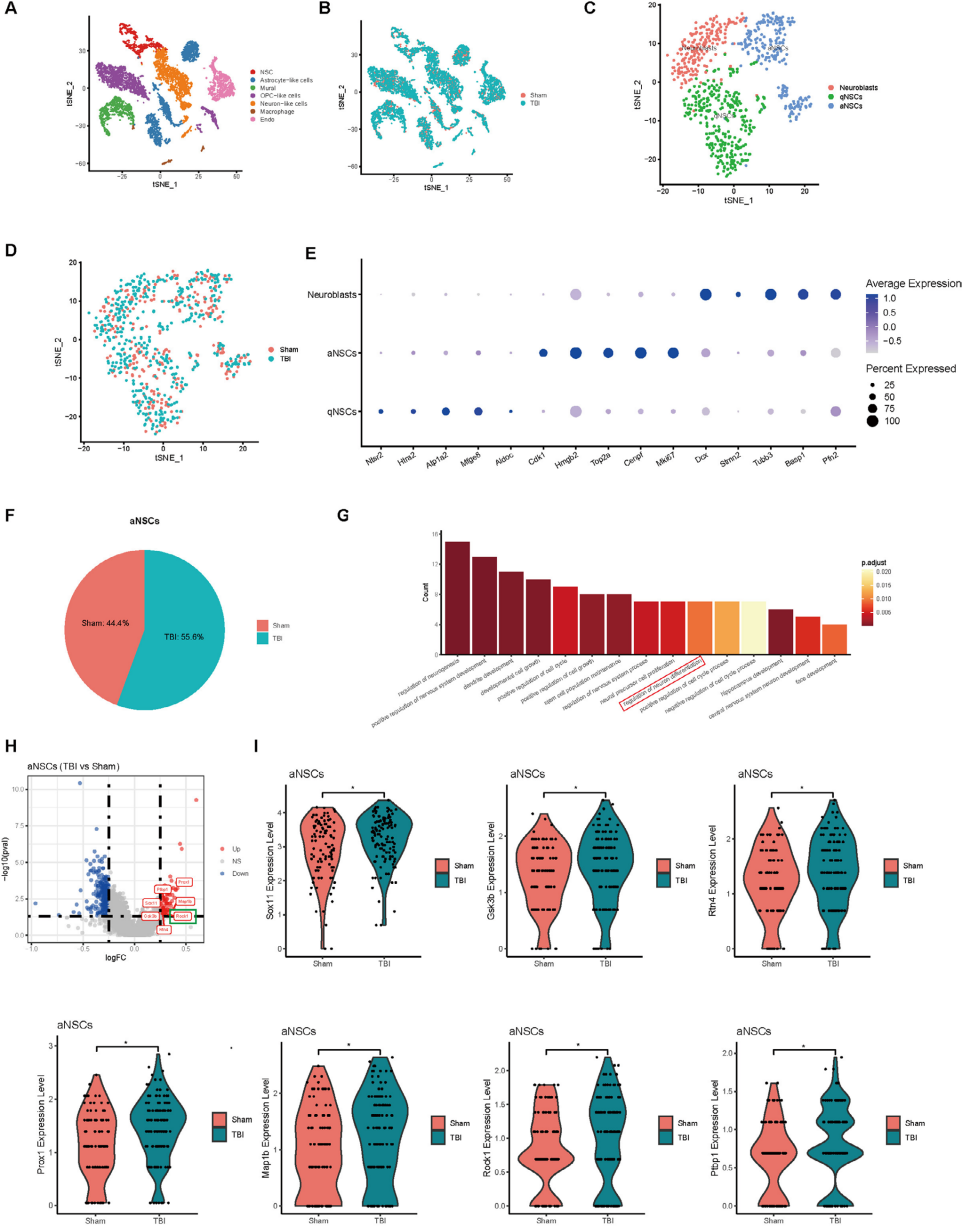
Single‐cell RNA sequencing analysis revealed upregulated expression of *ROCK1* in neural stem cells of TBI mice. (A, B) Perform t‐SNE dimensionality reduction on all cells, visualise the reduced‐dimensional space, and annotate cell types using known markers. (C, D) Perform t‐SNE dimensionality reduction on the neural stem cells, visualise the reduced‐dimensional space, and annotate the cell types based on known markers. (E) The expression of marker genes in neural stem cells at different stages. (F) Pie chart shows more aNSCs in the TBI group than in the Sham group, indicating that neural stem cells are activated after TBI. (G) Gene Ontology (GO) enrichment analysis of biological processes associated with the nervous system in aNSCs across experimental groups. (H, I) DEGs associated with the neuronal differentiation pathway and their expression patterns.

To explore the underlying mechanism of abnormal hippocampal neurogenesis following TBI, we performed scRNA‐seq analysis using mouse hippocampal tissues. Following the standard workflows, we conducted dimensionality reduction and unsupervised clustering on all cells, then visualised and annotated distinct cell populations (Figure 3A,B) [2]. Subsequent sub‐clustering of NSCs revealed heterogeneous subtypes (Figure 3C,D), which were further classified using stage‐specific markers (Figure 3E; Figure S1A) [26, 27]. By comparing the status of NSCs across various differentiation stages, we found that the TBI group exhibited a higher proportion of activated NSCs than the sham group (Figure 5F), which was consistent with our previous findings. Differential gene expression analysis and Gene Ontology (GO) enrichment of activated NSCs showed significant alterations in pathways related to neurogenesis (Figure 5G). Focusing on the “regulation of differentiation” pathway, we identified key differentially expressed genes (DEGs) and visualised their expression patterns (Figure 5H,I). Next, we validated selected DEGs individually by RT‐qPCR, and confirmed that the *ROCK1* gene expression was significantly upregulated in the TBI group compared to the sham‐operated group (Figure S2B). Likewise, ROCK1 protein level was significantly increased in the TBI group compared to the sham‐operated group as detected by western blotting (WB) (Figure S2C,D).

### 3.4 Inhibition of ROCK1 Expression Reverses Abnormal Hippocampal Neurogenesis After TBI

**FIGURE 4:**
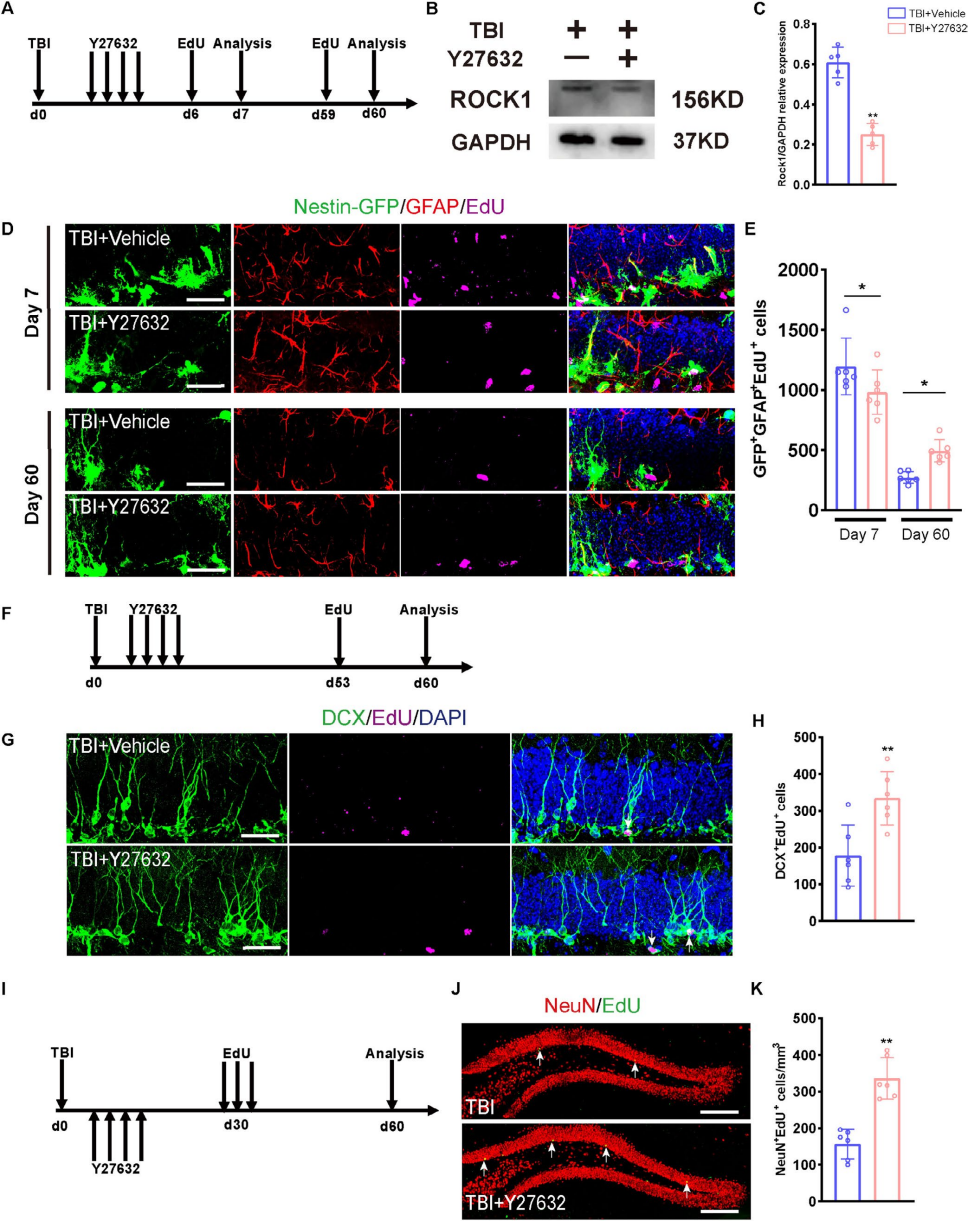
The ROCK1 inhibitor Y‐27632 attenuates neurogenesis impairment in the DG of the hippocampus post‐TBI. (A) Experimental timeline for cell proliferation analysis in the TBI group with or without Y‐27632 treatment. (B, C) ROCK1 and GAPDH protein expression levels in NSCs from the TBI + Y‐27632 and TBI + Vehicle‐treated groups. Semi‐quantitative analysis of WB results. GAPDH served as the internal loading control (*n* = 5). (D) Representative images of brain sections immunostained for GFAP, labelled with EdU, and showing genetically expressed GFP in the DG of TBI mice at 7 and 60 days post‐injury with or without Y‐27632 treatment. Scale bar = 20 μm. (E) Quantification of proliferating rNSCs (GFP^+^GFAP^+^EdU^+^) in group D (*n* = 6). (F) The experimental timeline for EdU administration and cell differentiation analysis of TBI group with or without Y‐27632 treatment. (G) Representative fluorescence micrographs of brain sections immunostained for DCX and labelled with EdU in the DG of TBI mice at 60 days post‐injury, with or without Y‐27632 treatment. Scale bar = 20 μm. (H) Quantification of new‐born immature neurons (DCX^+^EdU^+^) from E. (*n* = 6). (I) The experimental timeline for EdU administration and cell differentiation analysis of TBI group with or without Y27632 treatment. (J) Representative fluorescence micrographs of brain sections immunostained for NeuN and labelled with EdU in the DG of TBI mice at 60 days post‐injury, with or without Y‐27632 treatment. Scale bar = 100 μm. (K) Quantification of new‐born mature neurons (NeuN^+^EdU^+^) from J (*n* = 6). *p < 0.05, **p < 0.01, ***p < 0.001, Student's *t* test.

ROCK1 plays roles in a wide range of essential cellular functions, such as proliferation, adhesion, and migration. Altered ROCK1 expression has also been linked to tumorigenesis in cancers. However, it is still unknown whether ROCK1 expression has any role in NSC proliferation in the CNS. To inhibit the ROCK1 kinase activity, mice were i.p. injected with the ROCK1 inhibitor Y27632 for 4 consecutive days, starting from 24 h post‐TBI. Subsequently, EdU was administered on days 6 and 59 post‐TBI, and mice were euthanised 24 h after each injection (Figure 4A). WB analysis confirmed effective ROCK1 inhibition in the Y27632‐treated group (Figure 4B,C). Furthermore, immunofluorescence (IF) analysis displayed transient reductions in the population of GFAP^+^/GFP^+^/EdU^+^ proliferating rNSCs in Y27632‐treated mice on day 7 post‐TBI; however, this population of rNSC subtype was significantly increased on day 60 post‐TBI in the Y27632‐treated group compared to the sham group (Figure 4D,E). These results suggest that homeostatic regulation of ROCK1 protein level may prevent the overactivation of the NSC pool in the early stages of TBI pathology, thereby preserving a stable NSC pool. To assess the extent of differentiation in rNSCs, we performed an EdU pulse‐labeling via i.p. injection (50 mg/kg) at two critical phases –days 30 and 53 post‐TBI, followed by systematic perfusion‐fixation and brain tissue collection on day 60. Multilineage differentiation analysis of hippocampal rNSCs was performed by confocal microscopy (Figure 4F,I). Subsequent IF analysis revealed significant increases in the populations of DCX^+^/EdU^+^ and NeuN^+^/EdU^+^ rNSCs in Y27632‐treated mice compared to sham mice (Figure 4G,H,J,K), suggesting an enhanced survival or maturation of newly differentiated neurons by inhibiting ROCK1 expression after TBI.

**FIGURE 5:**
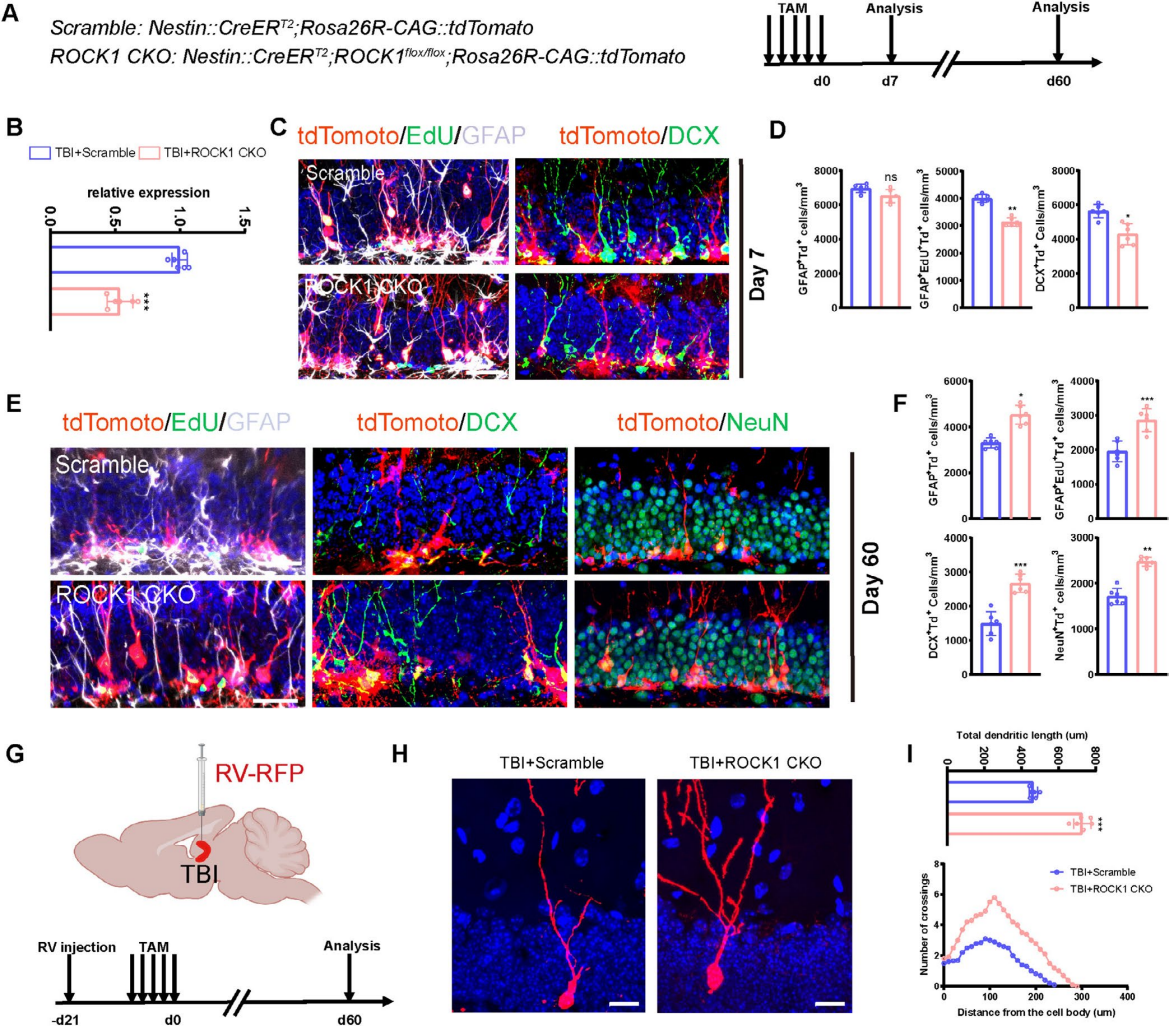
Conditional knockout of *Rock1* rejuvenates neurogenesis after TBI. (A) Schematic Diagram of ROCK1 Knockout and Experimental Timeline Design. (B) The RT‐qPCR results demonstrated successful knockdown of ROCK1. (C) Representative images of the DG in the TBI group and TBI + *ROCK1*‐CKO mice at 7 days post‐injury, showing proliferating NSCs (tdTomato^+^EdU^+^GFAP^+^ cells) and immature neurons (tdTomato^+^DCX^+^), demonstrating the lineage progression from neural stem cells to immature neurons. Scale bar, 20 μm. (D) Quantification analysis of tdTomato^+^GFAP^+^ cells, tdTomato^+^GFAP^+^EDU^+^ cells and tdTomato^+^DCX^+^ cells from C (*n* = 6). *p < 0.05, **p < 0.01; ns, not significant, Student's *t* test. (E) Representative images of the DG in the TBI group and TBI + *ROCK1*‐CKO mice at 60 days post‐injury, showing proliferating NSCs (tdTomato^+^EdU^+^GFAP^+^ cells), immature neurons (tdTomato^+^DCX^+^), and mature neurons (tdTomato^+^NeuN^+^). Scale bar, 20 μm. (F) Quantification analysis of tdTomato^+^GFAP^+^ cells, tdTomato^+^GFAP^+^EDU^+^ cells, tdTomato^+^DCX^+^ cells and tdTomato^+^NeuN^+^ cells from E (*n* = 6). (G) Schematic diagram of retrovirus injection and design of experimental time. (H) Representative images of retroviral RFP labeling in the DG of TBI mice and TBI + ROCK1 CKO mice 9 weeks post‐injection. Scale bar, 50 μm. (I) Quantification of the dendritic length dendritic complexity (*n* = 6). *p < 0.05, **p < 0.01, ***p < 0.001; ns, not significant, Student's *t* test.

To further explore the function of ROCK1 in adult DG‐NSCs and TBI‐induced neurogenesis, we conditionally knocked out (KO) the *ROCK1* gene in adult Nestin‐CreERT2::ROCK1^flox/flox^ mice (ROCK1 conditional knockout [CKO]) and traced the DG‐NSCs and their progeny with a Rosa26R‐CAG::tdTomato Cre‐reporter (Figure 5A,B). The results revealed that *ROCK1* CKO mice had fewer subpopulations of Td^+^/GFAP^+^, Td^+^/GFAP^+^/EdU^+^, and Td^+^/DCX^+^ NSCs on day 7 post‐TBI compared to the control group, while subpopulations of Td^+^/GFAP^+^, Td^+^/GFAP^+^/EdU^+^, Td^+^/DCX^+^, and Td^+^/NeuN^+^ NSCs were significantly higher on day 60 post‐TBI in the TBI group than in the sham group, suggesting that *ROCK1* CKO may attenuate early overactivation of NSCs, thus restoring the TBI‐induced impairment of hippocampal neurogenesis (Figure 5C–F). Additionally, injection of retroviral RFP vector in *ROCK1* CKO mice on day 60 post‐TBI exhibited longer total dendrite lengths and increased dendritic branch points in RFP^+^ newly differentiated neurons compared to controls (Figure 5G–J), suggesting that *ROCK1* CKO may enhance dendritic complexity and morphological maturation of hippocampal NSCs following TBI.

### 3.5 ROCK1 Inhibition Alleviates Cognitive Impairment After TBI

We demonstrated that ROCK1 inhibition could ameliorate abnormal neurogenesis in the hippocampus of TBI mice. Given the critical role of hippocampal neurogenesis in the maintenance of cognitive functions, we sought to investigate whether ROCK1 inhibition could improve TBI‐induced cognitive deficits. In the MWM test, ROCK1‐inhibited mice spent significantly more time in the target quadrant, walked higher numbers of platform crossings, and travelled longer path lengths in the target quadrant than sham mice (Figure S3A,B). Additionally, NOL and NOR tests showed significantly higher discrimination indices in ROCK1‐inhibited mice than controls (Figure S3C). These findings collectively indicate that ROCK1 inhibition may rescue TBI‐associated cognitive impairment.

### 3.6 ROCK1 Regulates Neurogenesis and Cognitive Function via AKT Phosphorylation After TBI

The AKT pathway is proposed to act downstream of ROCK1 [28]. While our previous studies demonstrate that the AKT/mTOR signalling plays a role in the proliferation of NSCs [29], whether ROCK1 modulates hippocampal neurogenesis post‐TBI via this pathway remains unknown. We observed that TBI significantly enhanced AKT phosphorylation in hippocampal NSCs (Figure S2D,E). Intriguingly, pharmacological inhibition of ROCK1 drastically suppressed AKT phosphorylation as well (Figure S2F,G), suggesting that altered ROCK1 expression may influence AKT activity in the post‐TBI hippocampus.

**FIGURE 6:**
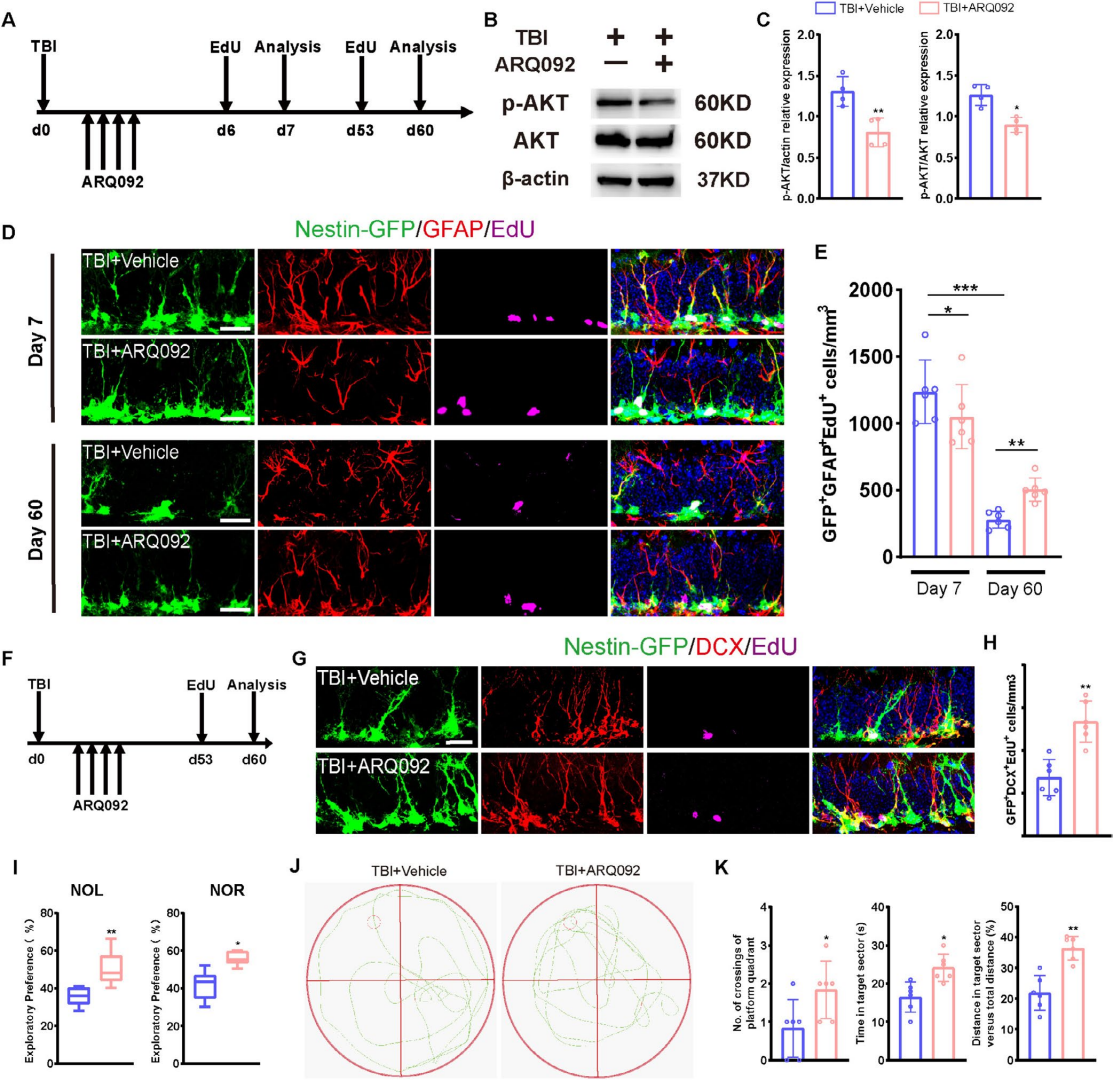
Rock1 affects hippocampal neurogenesis after TBI through upregulation of p‐AKT/AKT. (A) The experimental timeline for cell proliferation analysis of TBI group with or without ARQ092 treatment. (B, C) p‐AKT, AKT and β‐actin protein expression of NSCs from TBI + ARQ092 and TBI + Vehicle group. Semi‐quantitative analysis of WB results. β‐actin was served as the internal control(*n* = 4). (D) Representative images of brain sections immunostained for GFAP, labelled with EdU, and showing genetically expressed GFP in the DG of TBI mice at 7 and 60 days post‐injury with or without ARQ092 treatment. Scale bar = 20 μm. (E) Quantification of proliferating rNSCs (GFP^+^GFAP^+^EdU^+^) in group D (*n* = 6). (F) The experimental timeline for EdU administration and cell differentiation analysis of TBI group with or without ARQ092 treatment. (G) Representative fluorescence micrographs of brain sections immunostained for DCX, labelled with EdU and showing genetically expressed GFP in the DG of TBI mice at 60 days post‐injury, with or without ARQ092 treatment. Scale bar = 20 μm. (H) Quantification of numbers of new‐born immature neurons (GFP^+^DCX^+^EdU^+^) from G. (I) Pharmacological inhibition of AKT phosphorylation rescues TBI‐induced deficits in exploratory behaviour, as demonstrated by increased novel object exploration time in mice(*n* = 6). (J) Representative images of the swimming path of TBI + Vehicle and TBI + ARQ092 mice during the probe trial test in Morris water maze test at 60 days after TBI. (K) Behavioural performance of TBI mice with or without ARQ092 treatment in the Morris water maze: Escape latency during acquisition training; Time spent in the target quadrant during the probe trial; Number of crossings over the former platform location (*n* = 6). *p < 0.05, **p < 0.01, ***p < 0.001, Student's *t* test.

To explore the role of AKT in hippocampal neurogenesis of TBI mice, mice were administered a phosphorylated AKT (pAKT) inhibitor, ARQ092, for 4 consecutive days starting at 24 h post‐TBI. EdU was injected on days 6 and 59 post‐TBI, and mice were sacrificed 24 h later (Figure 6A). WB analysis confirmed significant downregulation of pAKT levels in ARQ092‐treated mice (Figure 6B,C). IF analysis consistently exhibited a lower population of GFAP^+^/GFP^+^/EdU^+^ proliferating rNSCs in the ARQ092 group than in the sham group on day 7, but significantly restored on day 60 (Figure 6D,E), suggesting that inhibition of pAKT might ameliorate the overactivation of NSCs, thereby preserving a stable pool of hippocampal NSCs. EdU was administered on day 53 to assess the population of immature neurons, followed by euthanasia on day 60 (Figure 6F). The result showed increased DCX^+^/EdU^+^ cell populations in ARQ092‐treated mice (Figure 6G,H). To identify mature neurons, EdU was administered on days 30–32 post‐TBI, with euthanasia on day 60 (Figure 6I). Notably, ARQ092‐treated mice exhibited significantly higher populations of NeuN^+^/EdU^+^ cells (Figure 6J,K), indicating improved survival or maturation of hippocampal NSCs. These results demonstrate that inhibition of pAKT levels could ameliorate TBI‐induced dysregulation of neurogenesis in the hippocampus.

**FIGURE 7:**
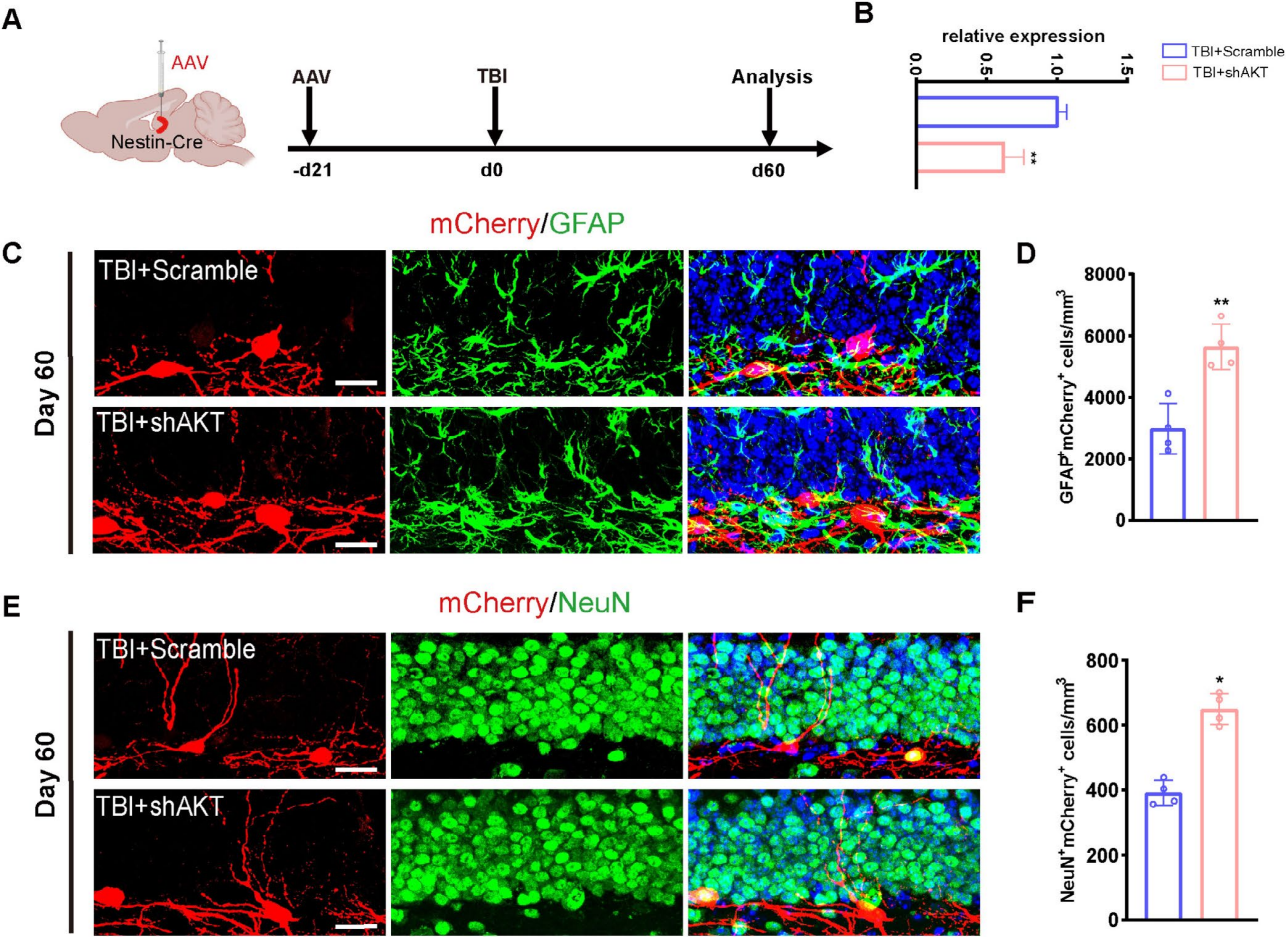
Conditional knockout of AKT rejuvenates neurogenesis after TBI. (A) Schematic design of the adeno‐associated virus injection and experimental timing. (B) The RT‐qPCR results demonstrated successful knockdown of AKT (*n* = 4). (C) Sample images of virus‐labelled mCherry cells co‐stained with GFAP in the adult DG. Scale bar, 10 μm. (D) Quantification of numbers of GFAP^+^mCherry^+^ cells from E (*n* = 4). (E) Sample images of virus‐labelled mCherry cells co‐stained with NeuN in the adult DG. Scale bar, 10 μm. (F) Quantification of numbers of NeuN+mCherry+ cells from G (*n* = 4). *p < 0.05, **p < 0.01, Student's *t* test.

To conditionally knock down AKT in mice NSCs, we injected AAV‐CMV‐FLEX‐mCherry‐AKT‐MCS‐WPRE or CMV‐FLEX‐mCherry‐MCS‐WPRE vector carrying a lox‐stop‐lox cassette into the hippocampus of Nestin‐Cre mice. Cre recombinase excised the stop cassette, enabling AKT CKO specifically in NSCs. Viruses were allowed 21 days for expression prior to the TBI operation (Figure 7A). Efficient AKT CKO was confirmed on day 21 post‐injection (Figure 7B). On day 60 post‐TBI, AKT CKO mice showed increased populations of hippocampal GFAP^+^/mCherry^+^ (Figure 7C,D) and NeuN^+^/Cherry^+^ (Figure 7E,F) NSCs compared to sham mice. These results demonstrate that TBI‐induced AKT upregulation contributes to aberrant hippocampal neurogenesis, and its inhibition restores neurogenic homeostasis.

Our previous results demonstrated that ARQ092, a pharmacological inhibitor of AKT phosphorylation, ameliorated TBI‐induced suppression of neurogenesis in the hippocampal DG. To explore whether inhibition of AKT phosphorylation improves cognitive function in TBI mice, we subjected ARQ092‐treated mice to a battery of behavioural tests. In the MWM test, ARQ092‐injected mice spent significantly more time in the target quadrant, frequently crossed platforms, and travelled longer distances than vehicle‐treated sham mice (Figure 6J,K). Furthermore, the NOL and NOR tests revealed higher discrimination indices in ARQ092‐treated mice than their sham controls (Figure 6I). These findings indicate that inhibition of AKT phosphorylation mitigates TBI‐induced cognitive deficits.

Both the ROCK1 level and the pAKT/AKT ratio play important roles in cell proliferation. We found that inhibiting ROCK1 resulted in a decreased pAKT/AKT ratio; however, the effect of inhibiting AKT phosphorylation remains to be determined. To explore the ROCK1‐pAKT axis, we measured ROCK1 protein levels in AKT phosphorylation‐suppressed models and found that modulating pAKT levels could not alter ROCK1 expression (Figure S2H,I). Together, the above results indicate that TBI‐induced ROCK1 upregulation leads to the exhaustion of NSCs, thereby impairing hippocampal neurogenesis by elevating pAKT levels. Therefore, targeting the ROCK1/AKT signalling axis in hippocampal NSCs could rescue the exhausted NSC pool, restore neurogenesis as well as protect cognition.

## 4 Discussion

The hippocampal brain region plays essential roles in cognition and behaviour as well as acts as a reservoir for brain stem cell niche, which is essentially involved in neurogenesis in the brain [30]. Although NSCs are essential in the adult brain neurogenesis and cognitive functions the specific contribution of NSCs to post‐TBI cognitive deficits remains unclear. In this study, we demonstrate that TBI‐induced upregulation of ROCK1 causes the overactivation of NSCs, leading to the exhaustion of the NSC pool and impaired neurogenesis under chronic conditions, ultimately resulting in irreversible cognitive dysfunctions. All experiments used young male mice (8 weeks) to control for hormonal and age‐related variability. While this approach enhances internal validity, we recognise the need to examine sex differences and aging effects in subsequent studies to establish broader applicability.

In the adult mouse brain, the majority of NSCs in the SVZ and DG niches remain quiescent under homeostatic conditions [7]. Upon activation, these dormant NSCs enter the cell cycle and initiate neurogenesis. Activated adult NSCs exhibit a dual function—self‐renewal to maintain their stemness properties and differentiation into mitotically active neural progenitor cells (NPCs) to expand the precursor population. These NPCs subsequently differentiate into neuroblasts and glial cells, thereby maintaining normal neurogenesis and ensuring homeostasis in the adult brain [6, 7]. While prior studies have observed that TBI skews NSC differentiation toward neurogenesis at the expense of astrogliogenesis, the temporal dynamics of astrocytic responses post‐TBI remain unexplored. Elucidating these time‐dependent changes could uncover novel therapeutic targets for TBI recovery. Under physiological conditions, resident NSCs in the adult mouse DG generate new neurons that integrate into the hippocampal circuits to support hippocampus‐associated learning and memory processes [31, 32].

Accumulating evidence suggests that TBI triggers neurodegeneration, leading to long‐term cognitive impairment and dementia [33, 34, 35]. Some studies have also demonstrated that hippocampal neurogenesis could be upregulated by TBI, with the number of proliferating NPCs peaking on day 7 post‐injury [2]. Notably, such persistent activation of NPC proliferation occurs in the DG niche of the hippocampus. However, there is a scarcity of information about the long‐term dynamics of neurogenesis in the NPC population following TBI, compounded by the absence of high‐resolution genetic lineage tracing techniques. Our study revealed that hippocampal NSCs marked by Nestin‐GFP expression in Nestin‐CreERT2 mice displayed heightened proliferative activity, specifically during the early phases of TBI. Furthermore, we identified persistent hippocampus‐dependent cognitive impairments in TBI models, which were consistent with observations from prior studies. In this study, we prioritised the Morris water maze (MWM) and novel object recognition (NOR) tests to evaluate traumatic brain injury (TBI)‐induced cognitive deficits. While TBI also triggers anxiety‐ and depression‐like behaviours, social interaction deficits, and substance use disorders in rodents, we deliberately excluded additional behavioural assays to minimise potential confounding effects from test battery‐induced stress. Prolonged behavioural testing may exacerbate fatigue‐related artefacts, compromising data validity. This represents a limitation of our current work; future studies will incorporate multidimensional behavioural profiling to address these broader neuropsychiatric dimensions.

Despite the severe consequences of TBI in patients, both specific therapeutic targets and effective pharmacological interventions for impaired neurogenesis remain critically limited [36, 37, 38, 39, 40]. To explore the mechanism of TBI‐associated impaired hippocampal neurogenesis, we conducted a single‐cell transcriptomics analysis, where we categorised all cells into seven clusters based on their reported cellular subtype markers [41, 42]. Next, we subdivided the NSCs into three groups—quiescent NSCs (qNSCs), active NSCs (aNSCs), and neuroblasts [26, 27]. These clusters represented the three distinct stages of NSC differentiation. Among them, aNSCs preferentially expressed genes encoding critical proliferation‐related factors. We further performed subgroup analysis and GO enrichment analysis related to neural cell development. The results suggest that ROCK1 may regulate neuronal differentiation processes following TBI. ROCK1 is a downstream effector of the small GTPase Rho and is activated through interactions with Rho family GTPases [43, 44]. ROCK1 is involved in fundamental cellular functions, such as proliferation, adhesion, and migration [45, 46]. While ROCK1 has been shown to promote tumour cell proliferation [47, 48, 49], its role in NSC proliferation remains enigmatic. Our results demonstrate that TBI‐induced ROCK1 upregulation could trigger aberrant overactivation of NSCs and subsequent depletion of the hippocampal NSC pool, while its inhibition effectively attenuates both hippocampal neurogenesis and cognitive deficits after TBI. Moreover, we labelled the morphology of mature neurons on day 60 post‐TBI to demonstrate that ROCK1 CKO could rescue TBI‐induced aberrant neuronal maturation.

The PI3K/AKT signalling acts downstream of the ROCK1 pathway. Studies have shown that AKT phosphorylation plays an important role in modulating neurogenesis [8, 12]. Here, we demonstrate that ROCK1 unidirectionally regulates AKT phosphorylation to influence neurogenesis. However, our findings did not elucidate the mechanistic paradox wherein the ROCK1‐AKT pathway promotes early‐stage activation of NSCs and drives late‐stage NSC depletion. Of note, we could not demonstrate whether the failure of activated NSCs to effectively differentiate into functional mature neurons could be attributed to either the depletion of the hippocampal NSC pool or impaired neuronal maturation. These unresolved questions will be prioritised in subsequent investigations.

In conclusion, our study reveals that pharmacological inhibition of ROCK1 (Y‐27632) and AKT phosphorylation (ARQ092) might rescue TBI‐induced neurogenic deficits, restore the NSC pool, and ameliorate cognitive dysfunctions. Although Y‐27632 and ARQ092 exhibit off‐target effects due to kinase structural homology and isoform cross‐reactivity, these limitations can be mitigated through targeted experimental design. Furthermore, these results provide a foundation for preclinical evidence supporting the hypothesis that targeted ROCK1‐AKT modulation could be a viable and potential therapeutic strategy for TBI patients.

## Author Contributions

J.W., H.G., and X.Z. designed and directed the study. C.Y., L.J., J.Z., Q.H., S.Z., C.W., H.L., J.Y., X.Y., Z.W., and N.D. performed the experiments. C.Y., L.J., and H.C. contributed to writing the original manuscript. J.Z. conducted the data analysis. Q.H. and Z.B. participated in confocal microscopy imaging. C.Y. and X.Z. prepared the illustrations. J.W., H.G., and X.Z. contributed to reviewing and editing the manuscript. All authors reviewed and approved the final manuscript.

## Conflicts of Interest

The authors declare no conflicts of interest.

## Supporting information


**Data S1.** Supporting Information.

## Data Availability

The data supporting this study were derived from the Gene Expression Omnibus repository, GEO accession GSE230942.
