# Correction to “Dysregulation of Rho‐Associated Coiled‐Coil Protein Kinase1 Depletes Neural Stem Cell Pool and Impairs Hippocampal Neurogenesis After Traumatic Brain Injury”

**DOI:** 10.1111/cpr.70206

**Published:** 2026-04-07

C. Ya, L. Jin, J. Zhong, et al., “Dysregulation of Rho‐Associated Coiled‐Coil Protein Kinase1 Depletes Neural Stem Cell Pool and Impairs Hippocampal Neurogenesis After Traumatic Brain Injury,” *Cell Proliferation* 50, no. 2 (2025 Aug 1): e70093, https://doi.org/10.1111/cpr.70093.

In Figure 1D, the image labelled ‘Day 14’ was incorrectly duplicated from ‘Day 28’.

The corrected Figure is reproduced below:

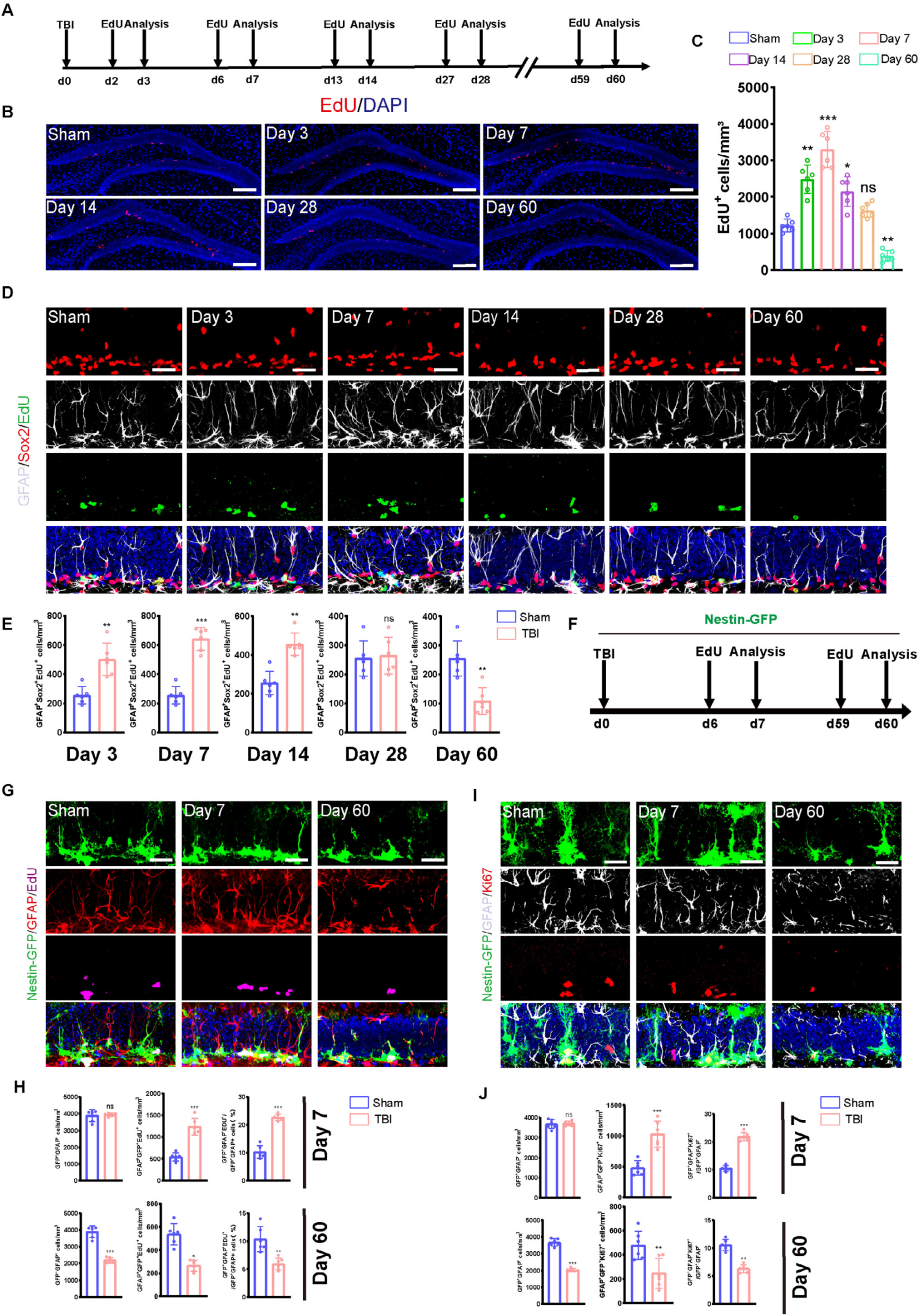

We apologise for this error.

