# Supplementary material for: Dysregulation of Rho‐Associated Coiled‐Coil Protein Kinase1 Depletes Neural Stem Cell Pool and Impairs Hippocampal Neurogenesis After Traumatic Brain Injury
Source: Cell Prolif. 2025 Aug 1;59(2):e70093. doi: 10.1111/cpr.70093 (PMC12877952; doi:10.1111/cpr.70093)
Supplement: Supplementary file 1 — Data S1. Supporting Information. [file CPR-59-e70093-s001.pdf]

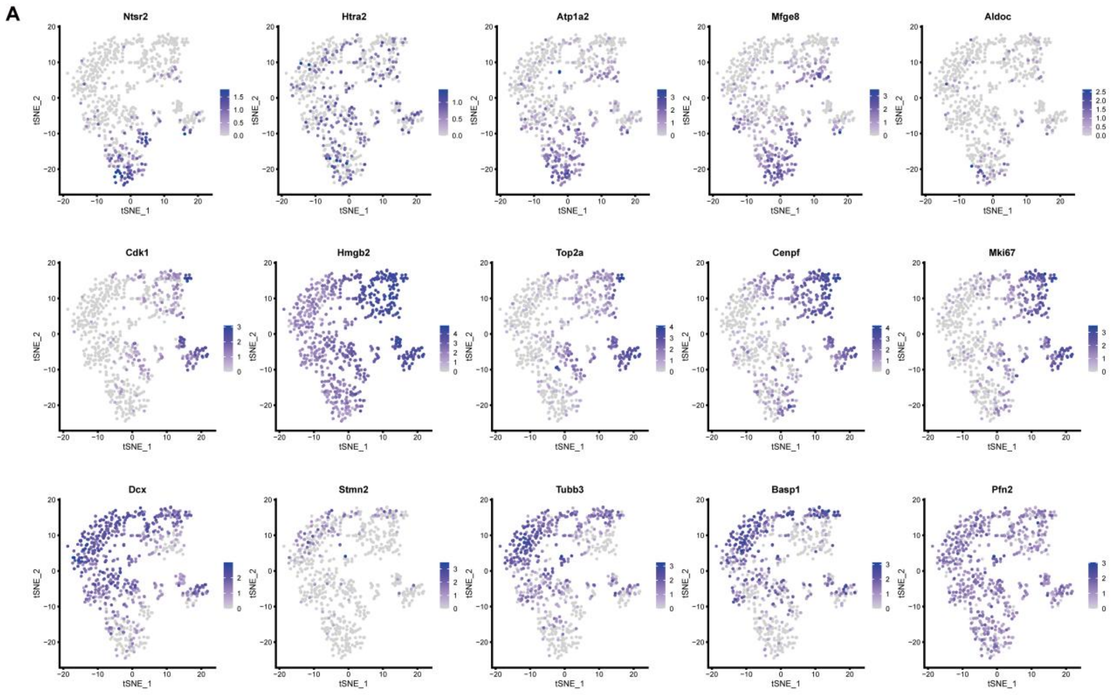

Figure S1. The expression of marker genes in neural stem cells at different stages.

A The expression of marker genes in neural stem cells at different stage

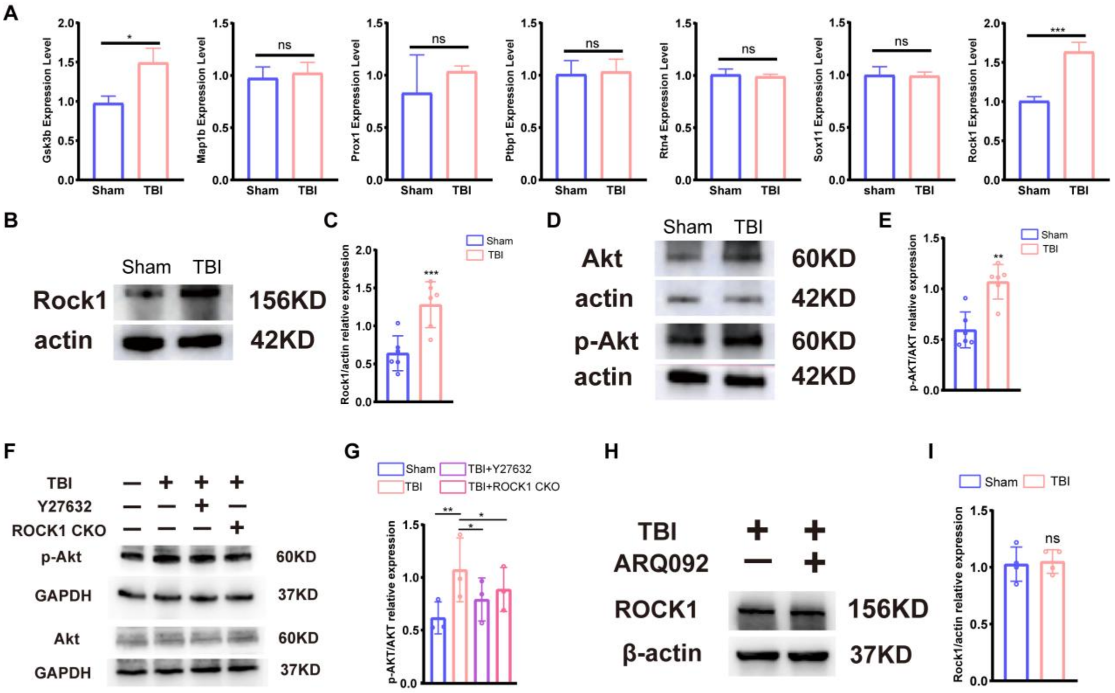

Figure S2. ROCK1 upregulates and regulates AKT phosphorylation post-TBI.

A Differential gene expression in the regulation of the neuron differentiation pathway was measured by RT-qPCR（n=6）.

B,C Rock1 protein expression of NSCs from TBI and Sham group. Semi-quantitative analysis of WB results. Beta-actin was served as the internal control（n = 6 ）.

D,E AKT, p-AKT protein expression of NSCs from TBI and Sham group. Semi-quantitative analysis of WB results. Beta-actin was served as the internal control(n = 6) . *p<0.05,**p<0.01, ***p<0.001; ns, not significant,Student's t test.

F,G Inhibition of Rock1 inhibited the phosphorylation of AKT, p-AKT protein expression of NSCs from Sham , TBI , TBI+Y27632 and TBI+ROCK1 CKO group. Semi-quantitative analysis of WB results. GAPDH was served as the internal control(n = 3) .*p<0.05； **p<0.01 . One-way ANOVA followed by Tukey's post hoc test.

H,I Rock1 and β-actin protein expression of NSCs from TBI+Vehicle and TBI+ARQ092 group. Semi-quantitative analysis of WB results. β-actin was served as the internal control.（n = 4 ）. ns, not significant,Student's t test.

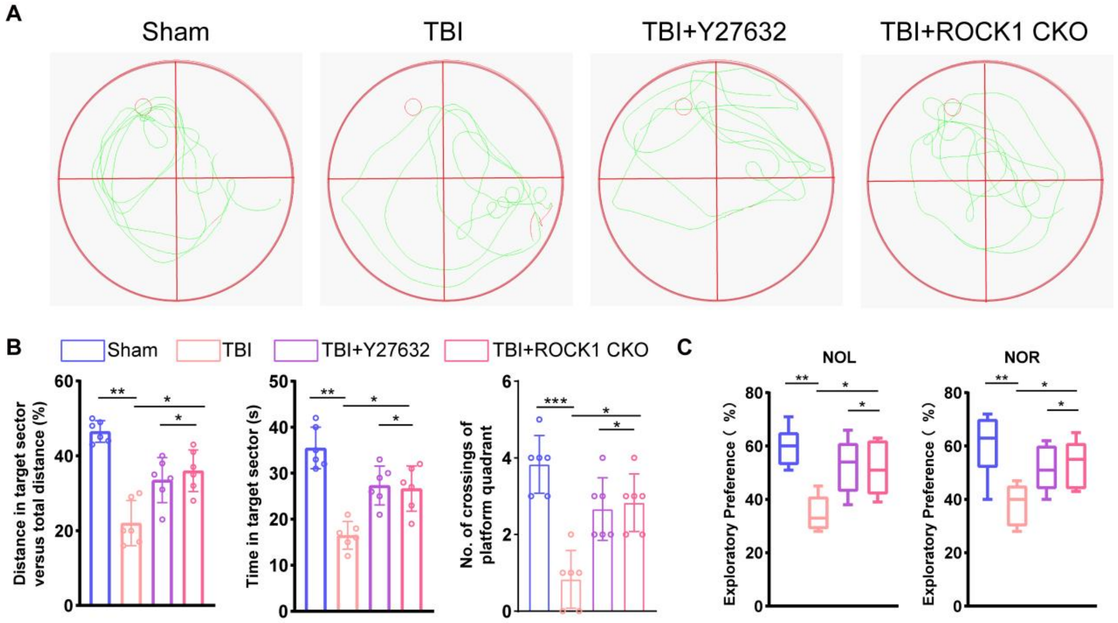

Figure S3. ROCK1 inhibition alleviates cognitive impairment after TBI.

A Mice with ROCK1 inhibition exhibited increased exploratory preferences in the Novel Object Location (NOL) test and Novel Object Recognition (NOR) test compared to TBI mice(n = 6) .**p<0.01.

B Representative images of the swimming path of TBI,TBI+Y27632 and TBI+Rock1 CKO mice during the probe trial test in Morris water maze test at 60 days after TBI.

C Behavioral performance of Sham-operated, TBI, TBI+Y27632 and TBI+ROCK1 CKO mice in the Morris water maze: Escape latency during acquisition training;Time spent in the target quadrant during the probe trial; Number of crossings over the former platform location (n = 6). *p<0.05； **p<0.01, ns, not significant . One-way ANOVA followed by Tukey's post hoc test.
